# COVID-19 and the impact of cash transfers on health care use in Togo

**DOI:** 10.1186/s12913-021-06895-2

**Published:** 2021-08-27

**Authors:** Yaovi Tossou

**Affiliations:** 1 Economics Department, University of Lome, Lome, Togo; 2 Member of the Research Centre for Economic Analysis of Public Policies (ANEPP), FASEG/University of Lome, Lomé, 01BP1515 Togo

**Keywords:** Cash transfers, COVID-19, Pandemics, Use of care, Propensity scores, Recipient households

## Abstract

**Background:**

Cash transfer program during pandemics provide a social protection mechanism to improve the health of the most vulnerable households. This article analysis the impact of cash transfers on household demand for health care during Covid-19.

**Methods:**

Using data from the survey conducted from 8th to 17th July 2020 covering all 44 districts in the 6 health regions of Togo under the direction of the United Nations Office for Project Services (UNOPS), we used propensity score matching and the ESR model. These models allow us to analysis the impact of cash transfers on health care use during Covid-19.

**Results:**

Analysis of the results shows a positive impact of cash transfers on the use of health care services in Togo for beneficiary households. In addition, the health insurance promotes the use of health care among households’ socio-economic factors.

**Conclusion:**

This cash transfer program is an effective approach to improving access to health care services for the most vulnerable households, particularly in the most disadvantaged settings. Thus, policy makers need to extend these cash transfers to a large part of the population during this Covid-19 health crisis as it has a positive impact on the demand for health care.

## Background

Cash transfers are a privileged tool for policy makers to help vulnerable populations bounce back in the face of shocks, particularly pandemics. It is also a common mechanism in humanitarian action, used to respond with greater dignity to people’s diverse needs. These transfers stimulate income-generating activities, help strengthen livelihoods and provide health care in the event of illness. In this way, cash transfer program during pandemics can make an important contribution to social protection and have significant health benefits [1]. They enable recipients to improve their health care choices. As such, they can be said to be an indicator for coping with health shocks for recipients as well as for non-beneficiaries. However, there are disparities within countries in the use of health care in sub-Saharan Africa. Thus, strategies for vulnerable households that focus on poverty reduction, social cohesion and access to health services are essential to improve health status and address health inequalities in sub-Saharan Africa [2]. Thus a promising and widely used intervention that could help households is cash transfers.

In addition, remittances represent risk management strategies for the most vulnerable households. From a family rationality perspective, these transfers can be more akin to spatial risk diversification than to maximizing expected economic returns. In such cases, they constitute insurance against cyclical hazards such as unemployment, illness, etc., particularly in countries where institutional insurance solutions do not exist.

Faced with these channels of transmission, cash transfers have served households in reducing the shocks caused by the COVID-19 pandemic. On one hand, these transfers enabled households to pay for COVID-19 control tools such as masks, disinfectants and hydro-alcoholic products, to self-medicate or to use health care services. On the other hand, the recipients of these transfers can act at different levels, ranging from increasing household purchasing power, to protecting productive assets, to improving household living standards. However, the socio-economic status of households is one of the determining factors in the use of health care services in the event of illness. It is affected by factors in terms of both demand and supply of health care. In terms of demand, many results show differences in the use of care by households during the COVID-19 pandemic. They are an integral part of demand-side health financing mechanisms. Conditional cash transfer program increased the use of free preventive health care services, but had variable effects on immunization coverage and anthropometric and nutritional outcomes among children. These socio-economic factors are often assessed according to income, occupation, level of education, age, place of residence and social protection.

For example, the WHO, 2020 finds that COVID-19 may have an adverse effect on the mortality rate of patients living with non-communicable diseases, resulting in a delay in the detection of non-communicable diseases progressing to more severe forms of the disease. This is due to the non-use of health facilities by households during the COVID-19 pandemic. These economic shocks affect the health sector, which prevents households without the financial means to attend health center in the event of illness. Faced with this situation, cash transfers make it easier for beneficiary households to use health center and use medicines to prevent illness. As a result of the pandemic in Africa, various sectors of health systems in low- and middle-income countries have been threatened, including pharmacies and hospitals [3]. In addition, [4] showed the effect of the COVID-19 pandemic on the use of pharmacies in Africa, which became very pronounced. In addition, the drug supply chain and pharmaceuticals have been strongly affected by restrictive measures introduced by policy makers.

In Togo, after the implementation of various policies to mitigate economic shocks due to the COVID-19, policy makers adopted a cash transfer mechanism called “NOVISSI”. It is a cash transfer program aimed at supporting any eligible Togolese citizen who has lost income due to the adoption of the response measures against COVID-19. The program aims to provide the most vulnerable individuals and families with monthly financial support throughout the state of emergency. In addition, households that have difficulty accessing health center in case of illness for fear of being infected, have the financial means to make use of health care. Thanks to these cash transfers, beneficiary households use them to reduce the costs of care that are directly or indirectly linked to the demand for health care. This facilitates access to and use of different health services during the pandemic. In addition, the rate of accessibility in health facilities in Togo is very high and requires financial means for the recipient households to use the cash transfer despite a good number of households doing self-medication/traditional care. With the COVID-19, most households receiving cash transfers can go to both public and private health center. This is due to direct and indirect costs related to the demand for care in private health center. Hence the low attendance in both public and private health facilities.

The population’s access to health care services has remained stable since 2016 (71.4%) against a target of 77.3% despite new health policies such as the construction of health facilities; extensions to existing health facilities and other new health facilities that are not functional due to lack of equipment (Annual Performance Report, 2018). However, descriptive analysis of the data used according to health care use during COVID-19 shows that 61.41% of households practice self-medication followed by 21.20% who use health care services compared to a small proportion of 17.39% of households who visit public health care facilities. Despite this high accessibility of health care to the population, COVID-19 showed a low frequency in public health center where health care costs are low. In addition, the remittance program dedicated to the most vulnerable households was only accessible to a small proportion of beneficiaries. Among this proportion of households, 5.8% of surveyed households receiving these cash transfers used health care when they are ill.

However, gaps in health systems during COVID-19 in the use of health care require mechanisms such as cash transfers and social benefits. This allows the frequency of non-communicable disease health care to be increased. This leads to lower morbidity and mortality. From all of the above, there is a need to find elements of answers to the problems mentioned. In view of this situation, the main research question is as follows: What is the impact of cash transfer on health care use during COVID-19.

In order to be able to answer this research question, the specific objectives of this article are as follows:

- Identify socio-economic factors that explain household use of health care during COVID-19;
- Analysis the impact of cash transfers on household use of care during COVID-19.

On the policy makers’ side, this article helps to alert decision-makers to the implications of appropriate policies to avoid a significant increase in mortality rates and the non-use of health care services. The outline of this article is organized as follows, section 1 focuses on the methodology referring to the different analysis on propensity scores and the ESR model. Section 2 deals with the source of the data and the description of the statistical variables. Section 3 is divided into two parts: The first part deals with the results obtained on the determinants of health care use based on the matching of propensity scores and the different tests, while the second part focuses on the results of the impact of cash transfers on health care use. Finally, a discussion of the results is followed by a discussion of the implications of economic policies.

## Methods

In this methodological part, we first present the propensity score model, then the results of the propensity score estimation and finally the effects of cash transfers on household health care use during COVID-19. In addition, the model developed for this analysis focuses on two levels among households receiving cash transfers during COVID-19. Firstly, we will consider the households that receive a cash transfer and secondly, the effect of the cash transfer on health care use.

### Theoretical framework

The impact assessment method used in this article is the matching group method introduced by [5] to measure the effectiveness of medical treatment. According to the author, when a given treatment is assigned to certain people (treatment group), this is usually done in a non-random way. As a result, the estimation of the effects of this treatment may be affected by selection bias.

In general, the processed data we have at our disposal is not collected randomly. Thus, there is evidence of selection bias. In order to overcome this problem of selection bias, [6] proposed the matching technique or propensity score matching. These methods were recently developed by [7] under the name “Kernel Matching”. In a fairly general way, the method consists in estimating the following model:

1 $$ {Y}_i=f\left({X}_i,{D}_i\right)+{u}_i $$

Where for each individual i, Y is an outcome variable (health care use); X is a vector of control variables; D is a treatment variable whose effects are being assessed; which is represented here by background transfer and an unobserved variable that contains the effect of other factors that determine Y. Let *Y*_1_ be the care use that a household would make if it benefited from the cash transfer and *Y*_0_ be _the_ health care use of the same individual if it did not benefit from the cash transfer. The treatment variable D takes the value 1 if the individual is a recipient of the cash transfer and 0 otherwise. For a given household i, the health care use during the observed COVID-19 is as follows:

2 $$ {Y}_i={Y}_{oi}+{D}_i\left({Y}_{1i}-{Y}_{oi}\right) $$

### Estimation of propensity score

The matching method assumes that the difference between treated and untreated individuals is due to their individual characteristics and the effects of the treatment. By neutralizing the differences that may arise from individual characteristics, it will remain the effect of the treatment. Matching individuals on the basis of these variables is a procedure that is difficult to carry out [7]. In view of these difficulties, groups are matched, according to [6], on the basis of a score known as a “propensity score”, which refers to the probability that a person with given characteristics will be a beneficiary of health insurance. This score is defined as follows:

3 $$ P(X)={P}_r\left(D=\frac{1}{\mathrm{X}}\right) $$

The characteristics forming the X vector will be noted Xi for each individual i. The propensity score is established on the basis of the probabilities conditional on the characteristics of the individuals to be in the different states distinguished:

4 $$ {P}_i^{k;l}=\frac{\Pr \left({D}_i=k/{X}_i\right)}{\Pr \left({D}_i=k/{X}_i\right)+\Pr \left({D}_i=l/{X}_i\right)} $$

The estimation of the propensity score consists of using the information available on non-recipients in order to be able to construct for each beneficiary if he or she would not have received cash transfers. Once the propensity score estimate is determined, the probabilities thus found by the scores are used to weight the observations so that any selection bias can be corrected for a robust estimate of the impact of the transfers on health care use. Before estimating the propensity score it is first necessary to select the appropriate model and the variables to be contained in it. These variables should influence both the treatment and the variable of interest. A test is then carried out to verify that the balancing property of the explanatory variables D ⊥ X | P(X) is satisfied and that the matching can indeed be carried out on the propensity score. With regard to the choice of model, the propensity score can be estimated either using a logit model or a probit model. Either the following logit model for the estimation of the score:

5 $$ {P}_i^{k,l}=\frac{\exp \left[\sum \limits_i\left({\beta}_j{x}_i\right)\right]}{1+\exp \left[\sum \limits_i\left({\beta}_m{x}_i\right)\right]} $$

Where β are the coefficients of the variables Xi.

### Principles for estimating the impact of cash transfers on the use of health care by households during COVID-19

Estimating only the propensity score does not account for the estimate of the average effect of the treatment because the probability of observing two units with exactly the same propensity score value is in principle zero since this probability is a continuous variable [8]. Benefiting from a cash transfer is a random phenomenon and the outcome of treated individuals can be used to calculate the counterfactual. All this under the assumptions of conditional independence and common support. Thus, therefore ATT = ATE is the average effect of cash transfers which determines the average effect of health care use for households that have undergone treatment. In view of the above, the general form of the estimator of the average effect of cash transfers on health care use by treated households is:

6 $$ ATT=\frac{1}{N_1}\sum \limits_{D_i=1}\left[{Y}_i^1-\sum \limits_{D_j=0}{\omega}_{N_0}\left(i,j\right){Y}_J^0\right] $$

Where N0 (respectively N1) represents the number of individuals in the control group represented by Di = 0 (respectively the number of individuals in the treated group represented by Di = 1); ωN0 (i; j) is a weighting function representing the weight assigned to each untreated individual j used to construct the counterfactual for treated individual i. These weights are such that ωN0 (i; j) = 1.

### Empirical foundations of the model (ESR) endogenous switching regression

In order to be able to address and test the second objective, which is entitled the impact of cash transfers on health care use, the endogenous switching regression (ESR) model is considered to confirm the robustness of the propensity score results. The first model is used to determine the probability that households will be able to receive a cash transfer. The second model relates the impact of cash transfers on households with and without a cash transfer. This empirical framework combines two potential characteristics between households [9].

### Source and data

The data used in this study comes from a national household survey conducted from 8th to 17th July 2020 covering all 44 districts of Togo’s 6 health regions. In each district capital, a minimum of thirty households were included by a systematic random draw at two levels (district then household). In each household, the head of household, all consenting adults and children/adolescents (10–17 years) were surveyed. The method used in the sample size was calculated using a single proportion of the population with a confidence level of 95%. We assumed that 50% of the population self-medication, with a margin of error of 5%. The minimum sample size estimate is 384 participants. A non-response rate of 10% was anticipated, and the minimum number of participants was estimated at 422. With a sample size of 955, we have achieved a margin of error of 3%. This small sample size is due to the low proportion of beneficiaries of the cash transfers that the Togolese state put in place during this COVID-19 pandemic. This data collection is part of the study carried out by the African Centre for Epidemiological and Public Health Research and funded by the United Nations Office for Project Services (UNOPS). The data were collected based on three standardized questionnaires implemented on a dedicated Android application. The household questionnaire, administered to the head of household or his/her representative, provided information on the general characteristics of the household. Then the adult questionnaire was administered to each participant aged 18 and over who agreed to participate in the study and included socio-demographic characteristics, knowledge and practical attitudes towards the COVID-19. Finally, the child questionnaire was sent to subjects aged 10 to 17 years old and mainly included socio-demographic information on the children, their schooling and the activities carried out during the epidemic period.

### Analysis of the results

#### Descriptive analysis of the difference test

The difference test compares the averages of the two groups in order to infer a relationship between the treatment group and the control group. A difference test on observable characteristics of socio-economic factors was carried out to see the similarity between households in the two groups (control and treatment group). From Table 1, we see that the two groups are identical on a number of characteristics observed in the households that benefited from the cash transfer program during COVID-19. However, significant differences can be observed in certain variables such as job loss, gender and region. The results show a higher frequency of health care use in favor of the treated groups, i.e. households that received the cash transfer. The results analysis reflect only averages but do not present the actual effect of the cash transfers on health care use. The fact that there are still differences between the observable characteristics of the two groups biases this result. Matching by propensity scores is then relevant because it will provide more robust results because the biases are neutralized.

**Table 1:** Testing for differences between groups on observable characteristics

Variables	Control group	Treatment group	Difference t-student
Number of persons in charge	4.10	3.65	2.51^b^
Health insurance	0.17	0.08	4.08^a^
Marital status	0.85	0.91	−1.49
Education	1.44	1.32	1.27
Age	1.67	1.82	−2.56
Loss of employment	0.12	0.35	−5.03^a^
Gender	0.44	0.24	5.49a
Change of income	0.87	0.94	−3.33^b^
Region	2.73	0.69	20.08^a^
Use of care	1.57	1.36	1.49

^a^ Significant at 1%; ^b^ significant at 5%;

### Distribution of propensity scores between the two groups

**Fig. 1:**
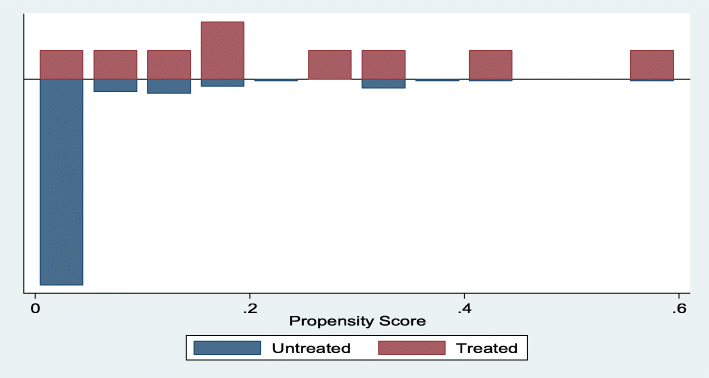
Distribution of propensity scores between the two groups

Figure 1 shows the results of the distributions of the common medium propensity scores which are defined over the interval [0.1; 0.6]. Analysis of this graph shows that the propensity scores have an overlapping distribution in the common support region for the treatment and control groups. This overlay shows that each treated individual (cash transfer recipient) can be matched to at least one control (non-recipient) individual. However, the lowest values of the propensity scores are around 0.3. The matching procedure will use very little data due to the lack of information in this area.

### Analysis of the distribution of propensity scores for health care use before and after matching

**Fig. 2:**
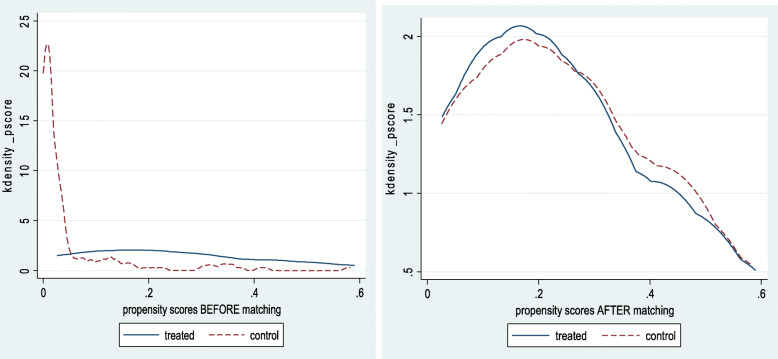
Analysis of cash transfer recipient households when using health care before and after treatment

Figure 2 analyzes a kernel density plot that estimates the underlying distributions of propensity scores before and after treatment or matching (intervention group; control group). Before matching, there is a substantial difference in the distributions of the two groups. After matching, the distributions of propensity scores are almost identical. This analysis shows the evolution of households receiving cash transfers due to COVID-19 or not. Before the treatment, the curve for the COVID-19 cash transfer recipient households (treated group) is spread to the right compared to the control group. This shows that households are likely to be able to use cash transfers to increase the frequency of health service use during the COVID-19 pandemic. After treatment, it is found that there is not much difference between the treated and control groups. The two curves are similar throughout the common support. It can be said that the matching between households receiving cash transfers and non-recipient households was successful.

### Analysis of propensity score distribution before and after treatment according to the characteristics of the variables

**Fig. 3:**
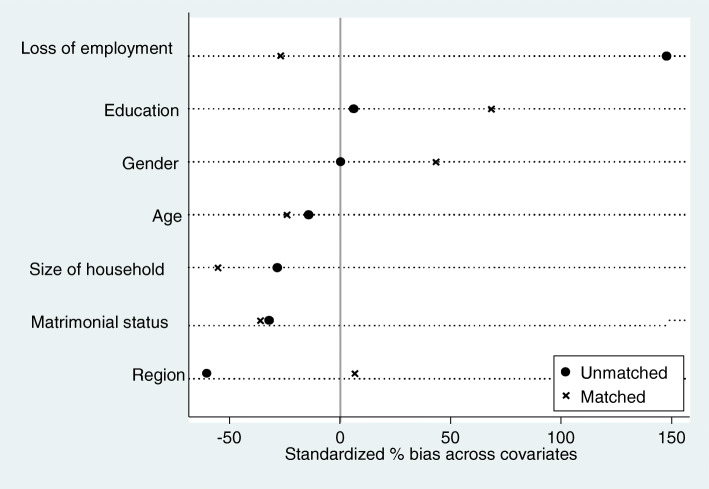
Analysis of the distribution of propensity scores before and after treatment according to the characteristics of the variables

Through this Fig. 3, the black dots show the success of the propensity scores, which correspond on average to 7 key covariates for the matching analysis. The black dots represent the average difference between the unmatched intervention and control groups at baseline. Cross dots represent the average difference between intervention and control group matched at baseline. Large initial differences are reduced to close to 0 by matching propensity scores between the two treatment groups. The results of the matching of the two groups according to their socio-economic characteristics show that prior to the matching, several items of the variables such as marital status, age, job loss, region are scattered between the treatment and control groups. After the treatment or matching, we find that some variables such as marital status are on the zero axis. On the other hand, variables such as education, household size are on the zero axis. All this shows that the treatment was successful. This confirms the analysis of the graph that the matching was carried out between the treated group and the control group. So the individuals are really similar and they have the same characteristics.

### Analysis of variable characteristics according to bias reduction and t-test

Table 2 shows the t-tests and the percentage of bias that occur when matching between the constructed variables. The matching of several variables such as job loss, marital status, level of education and height have a probability of more than 5%. This means that the matching has been carried out and that the individuals are all similar in terms of their socio-economic characteristics. We can say that households that did not have access to cash transfers during the pandemic are even likely to be recipients of cash transfers. Furthermore, the percentage of bias reduction for variable matching is very high for certain variables such as: loss of employment, marital status, level of education. This confirms the success of the matching of socio-economic variables.

**Table 2:** Analysis of variable characteristics according to bias reduction and t-test

Variables	Unmatched	Average		% of bias	% reduction of Bias	t-test		V(T)/ V(C)
	Matched	Treaty	Control			t	***p >*** t	
Gender	U	0.44	0.44	−0.5		− 0.01	0.20	0.31*
	M	0.44	0.22	43.3	− 9300	0.97	0.69	0.28*
Loss of employment	U	0.77	0.17	49.5		4.64	0.00	1.40*
	M	0.77	0.67	27.1	81.7	0.50	0.62	1.13
Age	U	1.67	1.74	−8.2		4.95	0.78	0.79
	M	1.67	1.33	38.6	− 370.0	−0.29	0.43	0.9
Marital status	U	0.67	0.86	−31.0		−1.06	0.29	–
	M	0.67	0.67	0.0	100.0	0.00	1.00	–
Region	U	1.56	2.55	−59.6		−2.00	0.05	1.15
	M	1.56	1	33.4	43.9	0.83	0.42	0.99
Education	U	1.67	1.59	7.2		0.22	0.83	0.9
	M	1.67	1.78	−11.4	−56.7	−0.21	0.83	0.88
Size of household	U	7.22	6.63	8.1		0.40	0.68	–
	M	7.22	0 6	16.8	− 106.8	0.36	0.76	–

### Impact of cash transfers on health care use

What emerges from the output of the results in the Table 3 is that we obtain a significant positive treatment of the impact of cash transfers on household health care use during Covid-19 on the treated group. This value is 0.666. This represents the rate of cash transfers received by the treated group is 66.6 points higher than the matched control group. Based on this analysis, we can say that cash transfers have a positive impact on health care use during these health shocks due to the Covid-19. This result confirms the fairly positive significance of the four tests carried out in the health insurance analysis.

**Table 3:** Impact of cash transfers on health care use

Variable	Sample	Treaty	Control	Difference	Standard deviation	t-stat
Use of care	Unmatched	1.33	1.53	0.19	0.27	−0.74
	ATT	1.33	2	0.67^a^	0.37	−1.79

^a^ significant at 10%

### Analysis of health care use outcomes: the ESR model

Assuming that there was a possibility of selection bias in the sample and the endogeneity that certain variables may present, any conclusions drawn from the analysis of the impact of cash transfers on health care use based on the estimation of the ESR model are likely to be biased. However, we have adopted endogenous switching regression (ESR) which eliminates observable and unobservable biases in the sample and provides a consistent estimate. The results in the table show that the effect of household socio-economic variables on cash transfers differs considerably between recipients and nonrecipients. The meaning of the term covariance noted *ρ*_*B*_ in the lower part of Table 4 shows the existence of an endogenous change between the selection equation and the use of care for households that have benefited from the cash transfer program. In other words, the meaning of *ρ*_*B*_ implies the presence of selection bias, which would have been a problem if it was not controlled in the cash transfer function by recipients.

**Table 4:** Results from the lower part of the ESR model

	Coefficient	t-student	***P >*** t
lns1	−0.52^c^	−1.79	0.07
lns2	−0.24^a^	−4.15	0.00
r1 (***ρ***_***B***_)	17.64^b^	1.85	0.09
r2 (***ρ***_***N***_)	−16.61^a^	14.4	0.00

^a^ Significant at 1%; ^b^ significant at 5%; ^c^ significant at 10%

In addition, the lower part of Table 4 deals with the estimation of the covariance terms. The terms of *ρ*_*B*_ and *ρ*_*N*_ have alternative signs, indicating that household participation in the cash transfer programme is based on a comparative advantage of the model. Thus, with *ρ*_*B*_ < 0 and significantly different from zero, implies that households that benefited from the cash transfer programme during the Covid-19 pandemic have higher than average health care use. Thus, they are more likely to participate in or benefit from a cash transfer program during the COVID-19 period. Similarly, with *ρ*_*N*_ > 0 and which is significantly different from zero, suggests that households receiving cash transfers with lower than average health care use are less likely to participate in a cash transfer program during COVID-19.

## Discussion

This study suggests that the cash transfer program put in place by policy makers during the COVID-19 pandemic increased the use of health care services for recipients. This program increased the demand for health care, which has a positive impact on households at this time of health crisis. These results are consistent with previous studies in other countries such as Mexico and Brazil, which also found greater effects of these transfers especially in rural areas [10–12]. The program has significantly increased the use of health services. For example, in the absence of financial incentives, households prefer unconventional alternative medicines such as traditional medicine. In addition, [13] have shown the effectiveness of cash transfer policies in improving children’s health, reducing the risk of morbidity, improving nutrition, increasing the use of health services and increasing immunization coverage. This program, called “NOVISSI”, provides a direct cash transfer to households that have lost all or part of their income due to the impact of the COVID-19 pandemic.

A number of studies have documented the direct effects of cash transfers on health status. A number of studies, such as the study on the direct effects of cash transfers on health status [14, 15], found a reduction in the prevalence of diarrhea among children whose parents received cash transfers. However, in Colombia, the results are not valid for children over 48 months of age. Our findings are consistent with those of [16, 17], which also indicate positive effects on the health status of children in cash transfer households compared to children of non-recipient parents.

In cash transfers, only households aged 18 and over benefited from these transfer mechanisms. However, in the use of health care, heads of households can use these cash transfers to pay for children’s health care. This has a positive effect on the use of care among children whose households benefit from cash transfers compared with children of non-recipient parents. In contrast, [17] show instead that households used their transfers to reduce the risk of high health care costs. In addition, they examine the impacts by age group (to children aged 0–5 years and to people aged 60 years) and find a positive impact of cash transfers on the health care use of both groups of individuals. This led us to conclude that there is a positive impact of cash transfers on the use of care also among children. Furthermore, the protective measures enacted by policy makers in terms of social distancing slow down all health service provision and the fear of attending clinics. It is very difficult for non-beneficiary households to access non-transferable health services. As a result, a decreasing number of households have access to health services.

### Limitations

The study shows the factors associated with the use of health care and the socio-economic inequality between recipients and non-recipients of cash transfers during this COVID-19 pandemic. However, this study has certain limitations. First, it focuses on households receiving money transfers that are located in urban areas. This did not allow us to study household behavior in the use of health care during this pandemic. In addition, the lack of information on household health care expenditures in the available data is also a limitation as it was not necessary to identify the catastrophic household spending during COVID-19. Furthermore, the study did not allow us to identify the socio-economic factors associated with the use of health care between recipients and non-recipients of cash transfers during this pandemic. COVID-19 in rural areas. The data collected only cover the 44 districts of the 6 health regions of Togo. Hence this distinction between rural and urban areas is not addressed. However, these mechanisms may be more beneficial for rural households, which have also suffered the health shocks of COVID-19. Furthermore, this limit should allow us to identify vulnerable groups of households that have benefited from this transfer of money and to analyze their behavior in relation to the use of health care. Finally, the limit is at the level of a number of beneficiaries of money transfers during this pandemic of COVID-19. Hence we have a small sample of the beneficiaries of this money transfer. Faced with these different limitations, we chose the socio-economic factors available in the database to conduct the analyze well and to find a good result. To overcome these limitations, the sample size must be high, covering different rural areas and the integration of key variables related to health care use.

## Conclusion

The objectives of this study are to analysis the impact of cash transfers on the use of care by households during COVID-19. Using the propensity score regression model and the ESR model, the results found show that cash transfers received by households during COVID-19 have a positive and significant impact on household health care utilization. The program has been successful in increasing the use of household health care services during the health crisis. In addition, factors such as health insurance, household education level, and place of residence are the socioeconomic factors that contribute to the use of health services during this COVID-19 pandemic. The results of this study suggest the formulation of economic policies in response to the COVID-19 health shocks as well as interventions that improve public health policies from a health care supply perspective.

Based on the survey data in Togo, the analysis carried out shows in general that household use of health care during the COVID-19 pandemic remains low. Only households that receive cash transfers are more likely to use health care services. In addition, the health insurance scheme is making a positive contribution to this health crisis. This is because households with health insurance facilitate their use by reducing the direct and indirect costs of health care. Hence a particular focus on health insurance coverage and cash transfers are mechanisms to reduce household care burdens in the event of a pandemics.

Thus, it would be interesting for policymakers to consolidate and expand social security mechanisms for the benefit of households so that they can be resilient to health shocks during the pandemic. In addition, policy makers need to establish a mechanism for financing unemployment insurance in the event of health shocks, the extension of family benefits to the informal sector and the establishment of a single social register. These different desired financing policies will enable households to have social protection to facilitate the use of health care in the event of pandemics.

## Data Availability

The data supporting the conclusions of this article are available at the African Centre for Research in Epidemiology and Public Health and funded by the United Nations Office for Project Services (UNOPS) of Togo data repository and can be obtained with a written permission. The datasets used and/or analyzed in this study are available from the Bioethics Committee for Health Research (CBRS) from the Togo Ministry of Health (CBRS No004/2020/CBRS) upon reasonable request. Data to support the findings in this article are available from CBRS and may be obtained with written permission. Data of this study are available without restriction. Contact to this Address: 89 rue des Sarrasins, BP 4089, Lomé Togo or on this address https://www.caresp-togo.org.

## Abbreviations

UNOPS: United Nations Office for Project Services
COVID-19: Coronavirus disease 2019
WHO: World Health Organization

## References

[1] Hansen AH, Halvorsen PA, Ringberg U, Førde OH (2012). Socio-economic inequalities in health care utilisation in Norway: a population based cross-sectional survey. BMC Health Serv Res.

[2] Owusu-Addo E, Renzaho AM, Smith BJ (2018). The impact of cash transfers on social determinants of health and health inequalities in sub-Saharan Africa: a systematic review. Health Policy Plan.

[3] Baldwin R, Tomiura E (2020). Thinking ahead about the trade impact of COVID-19. Economics in the Time of COVID-19.

[4] Akande-Sholabi W, Adebisi YA. The impact of COVID-19 pandemic on medicine security in Africa: Nigeria as a case study. Pan Afr Med J. 2020;35(73). 10.11604/pamj.supp.2020.35.2.23671. Accessed 10 Jun 2020. 10.11604/pamj.supp.2020.35.2.23671 PMC7875805 33623597

[5] Rubin DB (1974). Estimating causal effects of treatments in randomized and nonrandomized studies. J Educ Psychol.

[6] Rosenbaum PR, Rubin DB (1983). The central role of the propensity score in observational studies for causal effects. Biometrika..

[7] Heckman JJ, Ichimura H, Todd P (1998). Matching as an econometric evaluation estimator. Rev Econ Stud.

[8] Becker SO, Ichino A (2002). Estimation of average treatment effects based on propensity scores. Stata J.

[9] Lee L-F, Maddala GS, Trost RP (1982). Testing for Structural Change by D-Methods in Switching Simultaneous Equations Models. CENTER FOR NAVAL ANALYSES ALEXANDRIA VA NAVAL STUDIES GROUP.

[10] Lagarde M, Haines A, Palmer N (2007). Conditional cash transfers for improving uptake of health interventions in low-and middle-income countries: a systematic review. Jama..

[11] Barber SL, Gertler PJ (2009). Empowering women to obtain high quality care: evidence from an evaluation of Mexico’s conditional cash transfer programme. Health Policy Plan.

[12] Shetty A, Shetty S (2014). The correlation of health spending and infant mortality rate in Asian countries. Int J Contemporary Pediatr.

[13] Okereke M, Ukor NA, Adebisi YA, Ogunkola IO, Favour Iyagbaye E, Adiela Owhor G, et al. Impact of COVID-19 on access to healthcare in low-and middle-income countries: current evidence and future recommendations. Int J Health Plann Manag. 2020;36(1):13–7. 10.1002/hpm.3067. Accessed Jan 2021. 10.1002/hpm.3067 32857892

[14] Attanasio OP, Oppedisano V, Vera-Hernández M (2015). Should cash transfers be conditional? Conditionality, preventive care, and health outcomes. Am Econ J Appl Econ.

[15] Handa S, Peterman A, Seidenfeld D, Tembo G (2016). Income transfers and maternal health: evidence from a national randomized social cash transfer program in Zambia. Health Econ.

[16] Case A. Does money protect health status? Evidence from south African pensions. In: Perspectives on the Economics of Aging: University of Chicago Press; 2004. p. 287–312.

[17] Evans DK, Holtemeyer B, Kosec K (2019). Cash transfers and health: evidence from Tanzania. World Bank Econ Rev.

